# Cell-based analysis of *CLIC5A* and *SLC12A2* variants associated with hearing impairment in two African families

**DOI:** 10.3389/fgene.2022.924904

**Published:** 2022-08-11

**Authors:** Samuel Mawuli Adadey, Edmond Wonkam-Tingang, Leonardo Alves de Souza Rios, Elvis Twumasi Aboagye, Kevin Esoh, Noluthando Manyisa, Carmen De Kock, Gordon A. Awandare, Shaheen Mowla, Ambroise Wonkam

**Affiliations:** ^1^ Department of Pathology, Division of Human Genetics, Faculty of Health Sciences, University of Cape Town, Cape Town, South Africa; ^2^ Department of Biochemistry, Cell, and Molecular Biology, West African Centre for Cell Biology of Infectious Pathogens (WACCBIP), University of Ghana, Accra, Ghana; ^3^ Department of Pathology, Division of Haematology, Faculty of Health Sciences, University of Cape Town, Observatory, Cape Town, South Africa; ^4^ McKusick-Nathans Institute and Department of Genetic Medicine, Johns Hopkins University School of Medicine, Baltimore, MD, United States

**Keywords:** CLIC5, SLC12A2, hearing impairment pathobiology, Africa, hearing impairment

## Abstract

We have previously reported *CLIC5A* and *SLC12A2* variants in two families from Cameroon and Ghana, segregating non-syndromic hearing impairment (NSHI). In this study, biological assays were performed to further functionally investigate the pathogenicity of *CLIC5* [c.224T>C; p.(L75P)] and *SCL12A2* [c.2935G>A: p.(E979K)] variants. Ectopic expression of the proteins in a cell model shows that compared to wild-type, both the *CLIC5A* and *SLC12A2* variants were overexpressed. The mutant CLIC5A protein appears as aggregated perinuclear bodies while the wild-type protein was evenly distributed in the cytoplasm. Furthermore, cells transfected with the wild-type CLIC5A formed thin membrane filopodia-like protrusions which were absent in the CLIC5A mutant expressing and control cells. On the other hand, the wild-type SLC12A2 expressing cells had an axon-like morphology which was not observed in the mutant expressing and control cells. A network analysis revealed that CLIC5A can interact with at least eight proteins at the base of the stereocilia. This study has generated novel biological data associated with the pathogenicity of targeted variants in *CLIC5A* and *SLC12A2,* found in two African families, and therefore expands our understanding of their pathobiology in hearing impairment.

## Introduction

Hearing impairment can be caused by the malfunction of any part of the auditory system. Disruption of the mechanical transmission of sound through the external and middle ear results in conductive hearing impairment (Nadol, 1978). The mechanical auditory stimulus is converted to an electrical signal by neuro-epithelial hair cells in the inner ear and conducted by spiral-ganglion cells (Nadol, 1978). Failure of this process in the inner ear results in sensorineural hearing impairment which may be due to damage of the cochlear or vestibulocochlear nerve (Brodie et al., 2018). Mixed hearing impairment occurs when the external/middle ear and the inner ear are damaged (Zwartenkot et al., 2014). Over 124 genes have been associated with non-syndromic hearing impairment (NSHI) (Van Camp and Smith, 2020) and many of these genes are known to encode proteins with unique functions in the inner ear which participate in the hearing process (Delmaghani and El-Amraoui, 2020). Among the hearing impairment genes expressed in the inner ear are *CLIC5A* and *SLC12A2* and we have recently identified variants within both genes which were associated with hearing impairment in two families from Cameroon and Ghana, respectively. These variants reside within regions of high evolutionary conservation across species, and we have previously reported, using *in silico* analysis, that these are potentially pathogenic variants (Wonkam-Tingang et al., 2020; Adadey et al., 2021).

The *CLIC5* gene encodes for a protein which belongs to the Chloride Intracellular Ion Channels (CLICs) family of chloride ion channels that are unrelated to the major chloride ion channels (CIC) family. The CLIC family of proteins are particularly interesting for their dimorphism; they exist as either membrane-bound or soluble cytosolic proteins (Harrop et al., 2001; Gururaja Rao et al., 2020). They are characterized by approximately 241 amino acids with two GST-binding domains: one at the N-terminus, and the other at the C-terminus (Gururaja Rao et al., 2020). CLIC5 is an interesting member in that an alternative splicing produces two forms with different molecular masses; CLIC5A (251 amino acids) and CLIC5B (410 amino acids) of which their 238 amino acid C-terminal residues are identical to the core CLIC sequence. CLIC5A has a 13 amino acid N-terminal extension while CLIC5B has a 172 amino acid N-terminal extension. CLIC5A was observed to be present in solution as a dimer (Berryman et al., 2004) and is the predominant form expressed in hair bundles.

SLC12A2 belongs to the solute carriers (SLC) group which are transmembrane proteins that control the transport of molecules across the cell membrane. The SLC group of proteins are the largest group of transport proteins with more than 458 members belonging to over 65 distinctly described families (Cannizzaro et al., 2019; Pizzagalli et al., 2021). Though SLC proteins are mostly known for their membrane activity, some family members are found throughout cellular organelles (Pizzagalli et al., 2021). The SLC12 family, of which SLC12A2 is a member, consist of 9 proteins with a common structure of 12 transmembrane domains and intracellular N and C termini (Gagnon and Delpire, 2013). SLC12A2 is expressed in the inner ear and has been recently associated with hearing impairment in humans (Macnamara et al., 2019; McNeill et al., 2020; Morgan et al., 2020; Adadey et al., 2021).

In this study, we performed cell-based functional assays to pave the way for the understanding of the mechanism(s) by which the *CLIC5A* [c.224T>C; p.(L75P)] and *SCL12A2* [c.2935G>A: p.(E979K)] variants cause hearing impairment.

## Methods

### Site-directed mutagenesis

Mammalian expression constructs containing human *CLIC5A* (NM_001256023, UniProtKB - Q9NZA1) and *SLC12A2* (NM_001046, UniProtKB - P55011) cDNAs were purchased from ORIGENE (Rockville, Maryland). Both plasmids have GFP, myc, and DDK-FLAG tags as well as ampicillin and neomycin selection markers (Supplementary Figures S1A,B **)**. Site-directed mutagenesis (SDM) primer pairs (Supplementary Figures S1C,D) were designed for both plasmids according to the QuikChange Site-Directed Mutagenesis Kit (Stratagene) guidelines. The *CLIC5A* and *SLC12A2* mutant constructs were created using the KAPA Taq EXtra HotStart® ReadyMix™, amplified in *E. coli* cells and the *SLC12A2*: c.2935G>A and *CLIC5A*: c.224T>C mutations were confirmed using sanger sequencing (Inqaba Biotec, Gauteng, South Africa).

### Cell culture and transfection

The mutant or wild-type plasmids were transfected into HEK-293 cells (immortalized human embryonic kidney cells). HEK-293 cells were cultured in Dulbecco’s Modified Eagle Medium (DMEM) (Thermo Fisher Scientific, Massachusetts, United States) supplemented with 10% (v/v) fetal bovine serum (Thermo Fisher Scientific, Massachusetts, United States) and 1% (v/v) penicillin/streptomycin (Sigma-Aldrich, Missouri, United States), in a humidified incubator at 37°C and 5% CO_2_. Transfections were carried out using the X-tremeGENE™ HP Transfection Reagent (Sigma Aldrich, Missouri, United States) according to manufacturer’s instructions. In brief, 250 or 500 ng of the respective plasmid construct was mixed with the transfection reagent following the manufacturer’s protocol (pEGFP-N3 control, GFP-tagged wild-type CLIC5A and SLC12A2, or GFP-tagged mutant CLIC5A and SLC12A2) and used to transfect cells at a density of 4 × 10^4^ cells/ml or 8 × 10^4^ cells/ml for confocal microscopy or western blot experiments respectively.

### Total protein isolation and western blotting

Total protein was isolated using 2x Laemmli buffer (0.125 mM Tris-HCL pH 6.8, 4% SDS, 10% b-mercaptoethanol, 20% glycerol, and 0.005% of bromophenol blue) and boiled for 5 min at 95°C. Proteins were resolved on 8–12% SDS-PAGE gels and transferred to nitrocellulose membranes (Bio-Rad, Hercules, CA, United States). In addition to total protein quantification using The Bio-Rad QuantiPro BCA Assay kit, and loading equal concentrations on the SDS-PAGE gel, membranes were stained with Ponceau-S to verify adequate separation and equal loading and transfer of proteins. The membranes incubated in blocking buffer (1×PBS-Tween with5% (w/v) fat-free milk) for 1 h at room temperature followed by overnight incubation at 4°C in primary antibodies diluted in blocking buffer as follows: 1:1000 ANTI-FLAG® M2,(F1804, Sigma-Aldrich, Missouri, United States); 1:1000 anti c-Myc (9E10) (sc-40, Santa Cruz Biotechnology, Dallas, TX, United States), 1:5000 anti-p38 (M0800, Sigma-Aldrich Missouri, United States) and 1:5000 anti-p-p38 (Bio-Rad, Hercules, CA, United States)]. Secondary antibodies used were Goat Anti Rabbit (H + L) HRP conjugate (170–6515, Bio-Rad, Hercules, CA, United States) and Goat Anti Mouse (H + L) HRP conjugate (170–6516, Bio-Rad, Hercules, CA, United States) at 1:5000 dilution. In a dark room, the chemiluminescent substrate working solution (ECL Western Blotting Substrate from Thermo Fisher, Waltham, MA United States) was prepared following the manufacturer’s instruction and added to the blot and allowed to react for 5 min. The blot was removed and placed between clear plastic protector sheets and all bubbles removed. The blot was overlaid with photographic film and secured in a cassette. The film was developed and fixed in their respective solutions. The developed photographic film was air-dried and scanned for the densitometric analysis of signal intensity of the bands using ImageJ software (NIH, United States).

### Confocal microscopy

Live HEK-293 cells transfected with the appropriate plasmids viewed under Zeiss LSM8800 Airyscan confocal microscope (Zeiss, Oberkochen, Germany) 48- and 72-h post transfection. The nucleus was stained with 1:2000 of 10 mg/ml stock Hoechst stain (Invitrogen by Thermo Fisher Scientific, United States). The confocal microscope was equipped with a photomultiplier tube (PMT) detector which was able to detect the green fluorescence signal from a 488 nm Argon laser. The images were acquired and analyzed using ZEN Blue Software from Zeiss.

### In silico analysis of protein-protein interactions

A literature search was conducted (Supplementary Material S1) to identify publications on CLIC5A and SLC12A2 protein-protein interactions. Proteins that have biological interaction with CLIC5A or SLC12A2 were used to build protein interaction networks on STRING (Franceschini et al., 2012). The STRING is a web-based tool that predicts protein-protein interactions based on physical and functional associations from computational prediction, knowledge transfer between organisms, and interactions aggregated from other primary databases. The first shell with not more than 10 interacting proteins, with a score of not less than 0.4 (the default score) was considered for further analysis.

## Results

The clinical and family history of hearing impaired patients with *CLIC5A*: c.224T>C; p.(L75P) and *SLC12A2*: c.2935G>A: p.(E979K) variants from Cameroon (Wonkam-Tingang et al., 2020) and Ghana (Adadey et al., 2021) was previously reported. The current study provides biological evidence on the pathogenicity of the reported variants using cell models and biological assays.

### Mutant CLIC5A and SCL12A2 proteins are more stable than wild-type counterparts

**FIGURE 1:**
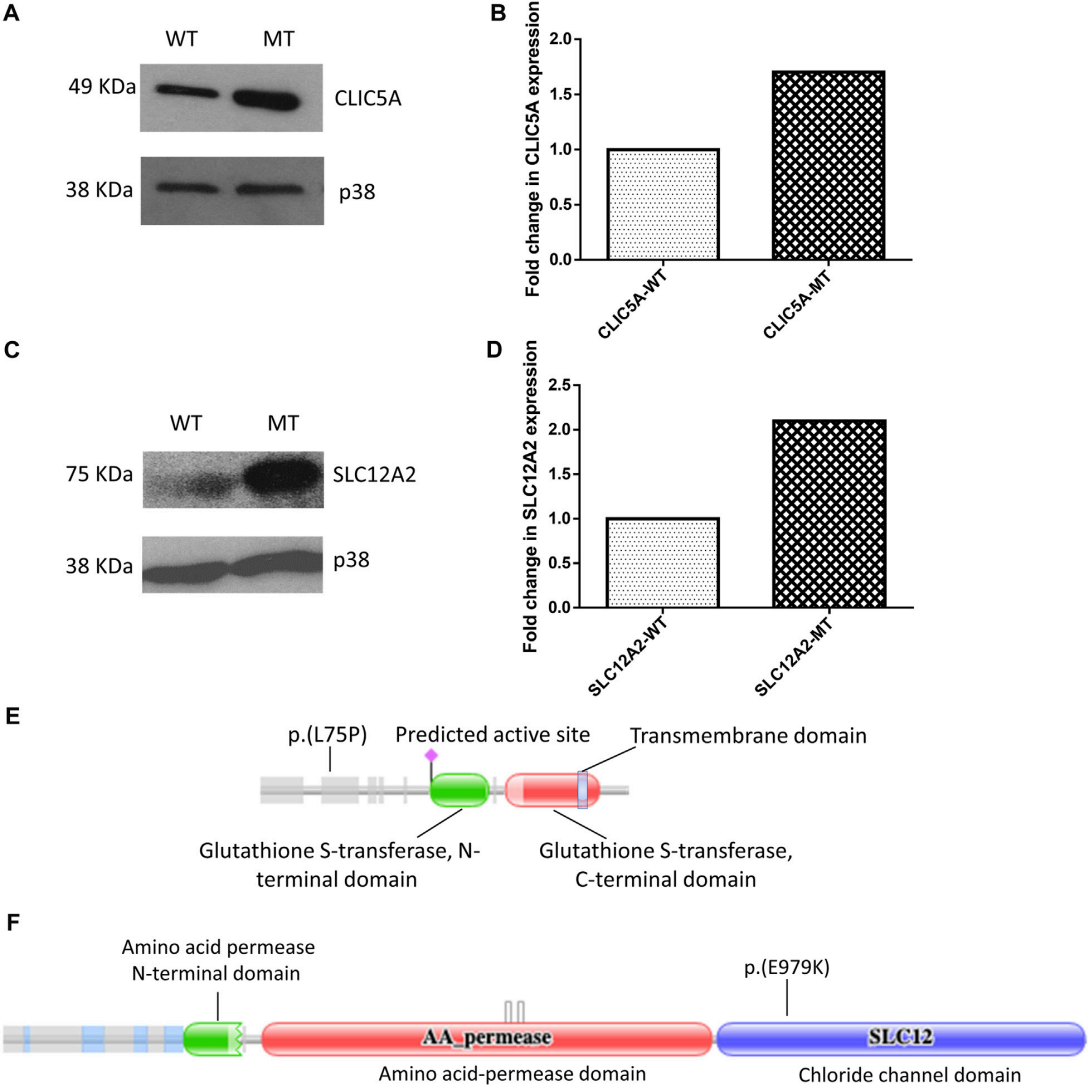
Mutant (MT) CLIC5A and SLC12A2 proteins are expressed at higher levels in HEK-293 cells, relative to the wild type proteins (WT). **(A)** Western blot of total proteins from HEK-293 cells transfected with wild-type (WT) or **(C)** 224T>C; p.(L75P) mutant (MT) *CLIC5A* plasmids; using anti-Myc antibody (N-terminal). **(B)** Densitometric analysis of western blot of CLIC5. **(C)** Western blot of total protein isolated from HEK-293 cells transfected with WT and **(C)** 2935G>A:p.(E979K) MT *SLC12A2* plasmids; using anti-flag antibody (N-terminal). **(D)** Densitometric analysis of western blots of SLC12A2 for the WT and MT SLC12A2 treated cells using ImageJ. The uncropped western blot pictures are shown in Supplementary Figure S3. Schematic diagrams showing **(E)** CLIC5 p.(L75P) and **(F)** SLC12A2 p(E979K) variant positions. The protein domains were predicted using the Protein Families Database (Pfam) (https://doi.org/10.1093/nar/gkaa913).

Western blot of total cell lysate of HEK-293 cells revealed differential expression of CLIC5A and SLC12A2 proteins when the mutant proteins were compared to the wild-type. It is worth noting that no statistical difference was observed between the transfection efficiency of the wildtype constructs compared to the mutant constructs (Supplementary Figure S2A). Notably, mutant CLIC5A and SLC12A2 proteins were in high relative abundance compared to wild-type CLIC5A and SLC12A2 proteins (Figures 1A,C). The densitometric analysis of the western blot gave a statistically significant difference (*p*-values of <0.0001 and 0.0006 for CLIC5 and SLC12A2) in the expression of the mutant and wild-type proteins with respect to the internal control, p38 protein (Figures 1B,C). The fold change in expression of the mutant protein was determined against the wilt-type protein. In brief, the ratio of wild-type densitometric readings against the internal control were set to 1 and used to compare that of mutant proteins. Although cell-based assays do not recapitulate *in vivo* occurrences, it is postulated that the above differences in protein levels possibly contributed to the hearing impairment phenotype by negatively impacting the protein function.

### Mutant CLIC5A protein is aggregated into distinct peri-nuclear inclusion bodies

**FIGURE 2:**
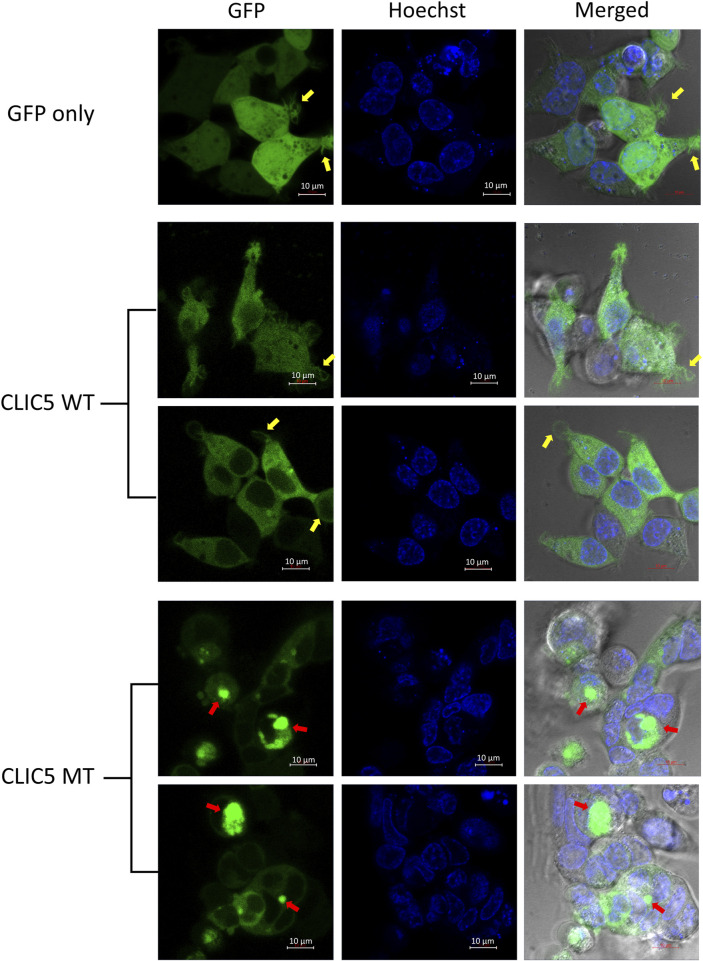
CLIC5A MT and not WT are aggregated into sub-cellular compartments. Live HEK293 cells expressing WT or MT GFP-tagged CLIC5A protein and stained with Hoechst were observed using confocal microscopy 48 h post transfection. The yellow arrows point to thin membrane filopodia-like protrusions observed in the WT transfected cells and the red arrows point to CLIC5 MT protein clusters.

Live cell imaging using confocal microscopy was used to analyze differences in protein localization and cellular morphology of GFP-tagged WT and MT proteins expressed in HEK-293 cells, with a hoechst stain used to stain the nucleus. As expected, control cells (GFP-only) displayed an even distribution of GFP throughout the whole cell (Figure 2). The wild-type CLIC5A protein was found to be evenly distributed throughout the cytoplasm and excluded from the nucleus. This was distinctly different to the mutant CLIC5A protein which appeared to aggregate within inclusion bodies found mostly in the perinuclear space (Figure 2, red arrows). With regards to cellular morphology, there were no major changes in cell shape observed between WT and MT CLIC5A, however a notable difference was that cells expressing wild-type CLIC5A had thin membrane filopodia-like protrusions (Figure 2, yellow arrows), which is similar to the control cells expressing GFP only, but this was absent in the cells expressing mutant CLIC5A.

### Cells expressing mutant SLC12A2 lose distinct morphological features and express less stress proteins

**FIGURE 3:**
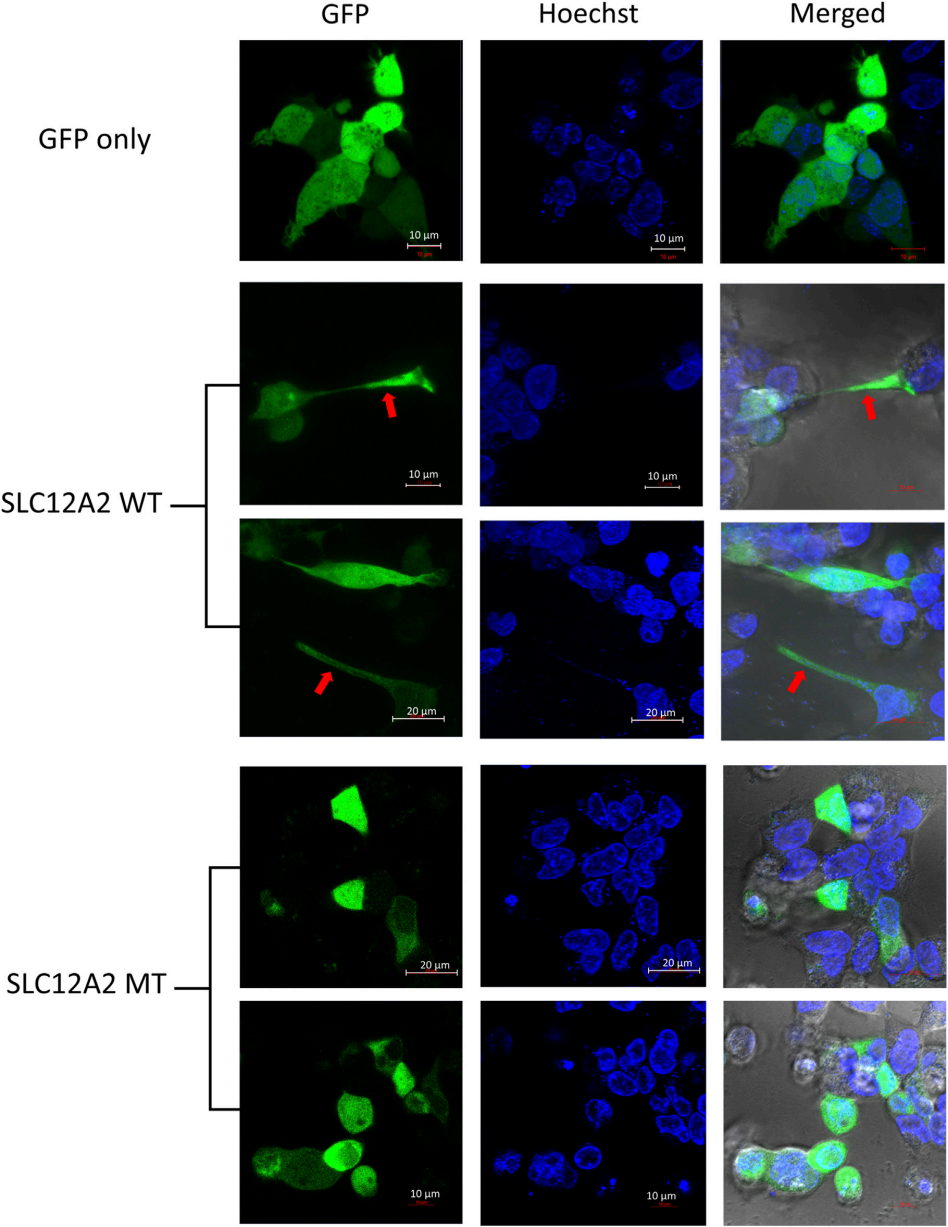
SLC12A2 WT HEK293 expressing cells display distinct morphology not observed in mutant-expressing cells. Live HEK293 cells expressing WT or MT GFP-tagged SLC12A2 protein and stained with Hoechst were observed using confocal microscopy 72 h post transfection. The red arrows point to axon-like structures present in the WT- SLC12A2 population of cells.

**FIGURE 4:**
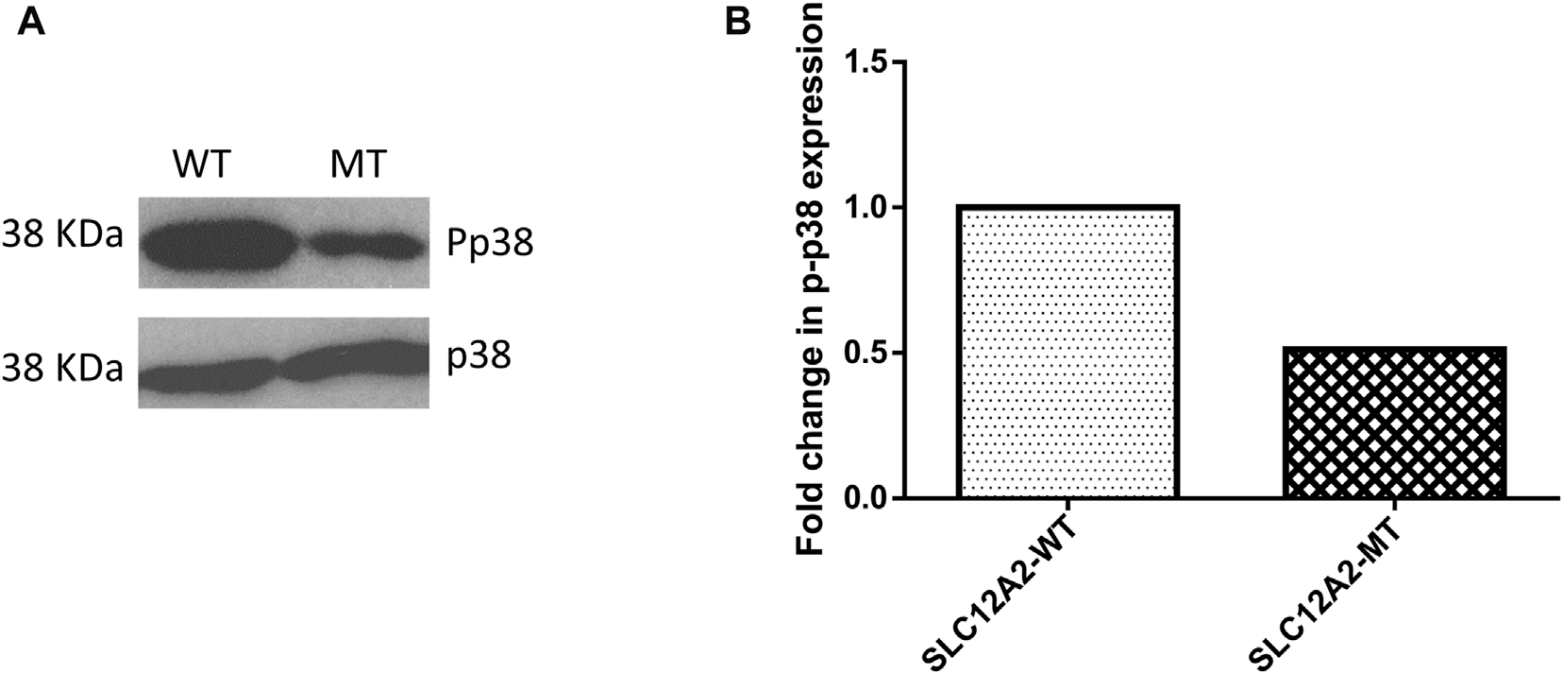
Mutant (MT) SLC12A2 expressing cells had relatively less phosphorylated p38 (Pp38) compared to the wild type (WT). **(A)** Western blot showing Pp38 expression in HEK293 cells transfected with WT and (C) 2935G>A: p.(E979K) MT *SLC12A2* plasmids. Total p38 was used for normalization **(B)** Densitometric analysis of western blots of Pp38 the WT and MT SLC12A2 treated cells using ImageJ. The densitometric analysis was conducted 3 times and the mean measurements were recorded. The uncropped western blot pictures are shown in Supplementary Figure S3.

The main difference observed among HEK-293 cells expressing GFP-tagged wild-type SLC12A2, when compared to mutant SLC12A2 was with regards to cell morphology. The Wild-type SLC12A2 cells displayed an elongated morphology and produced very distinct axon-like structures (Figure 3, red arrows) which were absent in the cells expressing mutant SLC12A2 or GFP only. Both wild-type and mutant SLC12A2 proteins were mostly localized to the cytoplasm of the HEK-293 cells (Figure 3). At 72 h post-transfection, it was observed that SLC12A2-expressing cells (both WT and MT) had detached and moved into suspension indicating stress on the transfected HEK-293 cells. Interestingly, the wild-type had relatively more detached cells than the mutant transfected cells. No cell detachment was observed in the mock-transfected, GFP-only transfected, as well as the CLIC5A-expressing cells at that time point. This prompted an investigation of the induction of cellular stress, which can be assessed via expression of stress pathway proteins (Coulthard et al., 2009). Indeed, expression of the stress phosphorylated p38^MAPK^ protein (p-p38) was significantly higher in WT- SLC12A2 expressing cells compared to those expressing MT-SCL12A2 (Figure 4).

### CLIC5A protein-protein interactions in the inner ear

**FIGURE 5:**
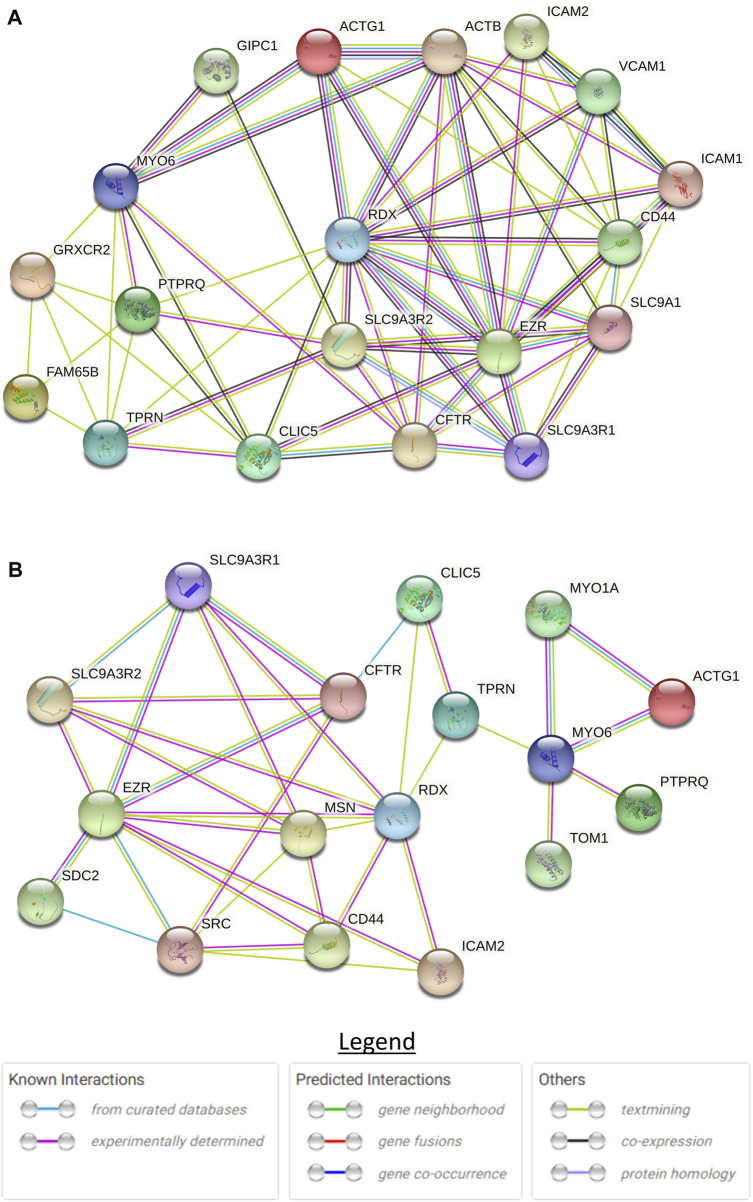
STRING protein-protein interaction network of CLIC5. **(A)** Full protein-protein interaction network consisting of both functional and physical protein interactions. **(B)** Subnetwork of physical interactions. The circles with structure in them are proteins with known or predicted structure.

To understand the role of CLIC5A in the inner ear, we investigated CLIC5A protein-protein interactions from published data and on the STRING database. Based on the experimental evidence from published literature, eight (8) proteins were found to interact with CLIC5A at the base of stereocilia (Supplementary Table S1). A protein network was built with these proteins which are known to be involved in the hearing processes or associated with deafness (Figure 5A). The protein network consisted of an additional ten (10) proteins predicted as functional partners (Supplementary Table S2). Most proteins on the network were either connected to EZR or RDX suggesting their major contributions to the formation of a protein complex at the base of stereocilia (Figure 5A). The CLIC5A-interaction network has shown that CLIC5A had physical interactions with only two out of the eight proteins, namely RDX and TPRN (Figure 5A), and an additional protein, CFTR. The physical interaction between CLIC5A and CFTR was from curated databases such as Biomolecular Interaction Network Databank (BIND), NCI-Nature Pathway Interaction Database (PID), Database of Interacting Proteins (DIP), and Biological General Repository for Interaction Datasets (BioGRID) (Figure 5B), however mice inner ear protein expression data suggests that CFTR may not be associated with HI.

### SLC12A2 protein-protein interactions in the inner ear

In the case of SLC12A2, no protein-protein interaction study was identified in the literature. SIX1 and SIX4 proteins were however reported to bind to the promoter of *SLC12A2* and regulate its expression in the inner ear (Ando et al., 2005). Both *Six1* and *Six4* have similar patterns of expression and are expressed in the inner ear of mice. Malformation of different organs including the inner ear was observed in *Six1* ^*−/−*^ mice and hence showing the importance of these *Slc12a2* regulatory genes in the development of the auditory system (Ando et al., 2005). The three proteins (SLC12A2, SIX1, and SIX4) were analyzed on the STRING database and no protein-protein interaction was observed between the two SIX proteins and SLC12A2.

## Discussion

Bioinformatics tools have proven to be effective in predicting the possible pathogenicity of variants, however, their utility is limited in terms of providing definitive evidence for biological pathogenicity (Flanagan et al., 2010). Thus, animal and cell models are critical to elucidate the possible mechanisms of pathogenicity of variants and ascertain their association with human trait and diseases. In this study, investigating protein expression, localization, and morphology of HEK-293 cells, we have provided additional cell-based assays data to support the pathogenic effect of previously reported variants in *CLIC5A* [c.224T>C; p.(L75P)] and *SCL12A2* [c.2935G>A: p.(E979K)].

CLIC5A, the isoform expressed in the inner ear, was initially isolated from placental epithelial cell microvilli as a component of the actin-based cytoskeletal complex, interacting with actin and ezrin at the cell cortex (Berryman and Bretscher, 2000; Berryman et al., 2004). CLIC5A is also expressed in the apical membrane of kidney glomerular endothelial cells (Nyström et al., 2009), podocytes (Tavasoli et al., 2016a), and placental microvilli (Berryman and Bretscher, 2000; Berryman et al., 2004). It was later shown to be highly expressed in the cell stereocilia of the inner ear, and important for sensorineural hearing (Gagnon et al., 2006). Mice deficient of the protein exhibited impaired hearing and vestibular dysfunction (Gagnon et al., 2006). A literature search revealed that the first report of *CLIC5A* variants were in hearing-impaired individuals from Turkey (Seco et al., 2015), with a homozygous nonsense mutation [*CLIC5A*: c.96T>A: p.(C32∗)] in two affected individuals with NSHI from the same family. The second report was our previous study from Cameroon where we found compound heterozygous variants [*CLIC5A*: c.224T>C: p.(L75P) and c.63 + 1G>A] in three NSHI patients (Wonkam-Tingang et al., 2020).

In the present study, we found that the mutant CLIC5A protein was proportionally more abundant compared to the wild-type when ectopically expressed in HEK-293 cells and this indicates that the mutant protein likely lost a domain important in cellular turnover. *In silico* analysis of this variant showed that leucine at position 75 of the CLIC5A protein is highly conserved and vital to the protein structure and function (Wonkam-Tingang et al., 2020). The effect of the variant on the protein structure and stability probably resulted in a misfolded and/or non-functional protein which accumulate within autophagosomes or aggresomes when the capacity of the proteosome pathway is exceeded (Johnston et al., 1998). This was supported by the subcellular localization experiment, performed in the present study, which showed that the mutant CLIC5A protein aggregated in perinuclear structures while the wild-type protein was distributed in the cytoplasm of the cell. In the cell, CLIC5A is known to be an integral membrane protein and functions as an ion channel however, it also exists as a soluble cytosolic protein (Dulhunty et al., 2001; Harrop et al., 2001; Gururaja Rao et al., 2020) explaining the cytosolic distribution of the wild-type protein in the HEK-293 cells. During protein synthesis, which takes place in the endoplasmic reticulum (ER), it is possible that the mutation in the CLIC5 protein leads to aggregation, hampering its trafficking outside of the ER, and thus making it unavailable for its normal cellular function.

We observed thin membrane filopodia-like protrusions in the CLIC5A wild-type transfected cells which were absent in the mutant transfected cells as well as control cells. CLIC5A is known to play an important role in the assembly of F-actin-containing complexes, an ATP-dependent process, via the activation of ezrin-radixin-myosin (ERM) proteins in a phosphatidylinositol (4,5)-bisphosphate-dependent manner (Berryman et al., 2004; Tavasoli et al., 2016b). In the inner ear, CLIC5A colocalizes with radixin (RDX), taperin (TPRN) and myosin VI (MYO6) at the base of the stereocilia (Salles et al., 2014). RDX, protein tyrosine phosphatase receptor Q (PTPRQ), and TPRN which are associated with deafness, were mislocalized in fused stereocilia of CLIC5A-deficient mice (Salles et al., 2014). Additionally, CLIC5A was shown to be particularly important for the localization of PTPRQ, RDX, and TPRN in the hair cell rootlet (stereocilia base) and CLIC5A-deficient hair cells showed diffusion of PTPRQ, RDX, and TPRN. Therefore, the absence of thin membrane filopodia-like protrusions in the CLIC5A mutant transfected cells may be attributed to inadequate formation of actin-ERM complexes, known to stabilize the linkage between the plasma membrane and actin cytoskeleton, as a result of the dysfunctional mutant CLIC5A protein (Salles et al., 2014). It is however also possible that the absence of thin membrane filopodia-like protrusions in the mutant CLIC5 expressing cells is as a result of stress response due to the aggregation of the mutant proteins.

The formation of the actin multiprotein complex by CLIC5A, RDX, TPRN, MYO6, and PTPRQ was shown to be critical in membrane-cytoskeletal attachment at the base of the hair bundle, involving the positioning and maintenance of the stereocilia shaft (Salles et al., 2014). Glutaredoxin domain-containing cysteinerich protein 2 (GRXCR2) and RHO family interacting cell polarization regulator 2 (RIPOR2) were reported to interact and localize with CLIC5A, and are essential for normal hearing (Li et al., 2021). Disruption of the interaction between CLIC5A and GRXCR2 was found to have minimal effects on stereocilia morphogenesis, however, the disruption led to hearing impairment in mice (Li et al., 2021). This underscores the role of GRXCR2 in the formation of the complex and the function of the stereocilia. There are two models to explain the role of CLIC5A at the base of the stereocilia (Salles et al., 2014; Li et al., 2021). Although these models are essential in our understanding of the formation of the protein complex, major proteins were left out in each model. Our literature review and CLIC5A protein interaction network (Figure 4) suggested that at least 8 proteins are involved in the formation of this complex at the base of the stereocilia.

According to the literature search, *SLC12A2* variants were associated with both NSHI and syndromic HI. Our previous report from Ghana and Pakistan (Adadey et al., 2021) and a study from Japan (McNeill et al., 2020) reported variants in NSHI patients (Table 1). The Ghanaian variant (c.2935G>A: p.(E979K) was also found in a Japanese who had NSHI and vestibular areflexia syndrome (CANVAS) (McNeill et al., 2020). The other NSHI variants found were c.3431C>A: p.(T1144N) in Italy (Morgan et al., 2020) and c.2941G>T: p.(D981Y) in Japan. A non-synonymous variant and a splice site variant were reported in unrelated individuals with motor developmental delay (Table 1).

**TABLE 1:** Association of *CLIC5* and *SLC12A2* variants with hearing impairment in patients.

Gene	Nucleotide change	Protein change	Inh	GT	Condition	Country	References
*CLIC5*	c.224T>C	p.(L75P)	AR	Het	NSHI	Cameroon	Wonkam-Tingang et al. (2020)
*CLIC5*	c.63 + 1G>A	-	AR	Het	NSHI	Cameroon	Wonkam-Tingang et al. (2020)
*CLIC5*	c.96T>A	p.(C32^∗^)	AR	Hom	NSHI	Turkey	Seco et al. (2015)
*SLC12A2*	c.2935G>A	p.(E979K)	AD	Het	NSHI	Ghana	Adadey et al. (2021)
			AD	Het	congenital, severe HI	Japan	Mutai et al. (2020)
			AD	Het	NSHI with vestibular areflexia syndrome (CANVAS)	-	McNeill et al. (2020)
	c.2939A>T	p.(E980V)	AD	Het	NSHI	Pakistan	Adadey et al. (2021)
*SLC12A2*	chr5: 127441491–127471419 delins34	-	AR	Hom	HI with Kilquist syndrome	Mixed European ancestry	Macnamara et al. (2019)
*SLC12A2*	c.3431C>A	p.(T1144N)	AD	Het	NSHI	Italy	Morgan et al. (2020)
*SLC12A2*	c.2941G>T	p.(D981Y)	AD	Het	NSHI	Japan	Mutai et al. (2020)
*SLC12A2*	c.2930–2A>G	-	AD	Het	HI with minor motor developmental delay	Japan	Mutai et al. (2020)
*SLC12A2*	c.2962C>A	p.(P988T)	AD	Het	HI with motor developmental delay	Japan	Mutai et al. (2020)
*SLC12A2*	c.980C>T	p.(A327V)	AD	Het	HI with multiple congenital anomalies (iris coloboma, ventricular septal defect and tracheo-oesophageal fistula)	United Kingdom	McNeill et al. (2020)
*SLC12A2*	c.555dupG	p.(H186 Afs^∗^17)	-	-	Spastic quadriparesis and severe global developmental delay	United Kingdom	McNeill et al. (2020)
*SLC12A2*	c.1127A>T	p.(N376I)	AD	Het	Spastic paraparesis and delay of speech and gross motor development	United Kingdom	McNeill et al. (2020)
*SLC12A2*	c.1135_1136del GCinsCT	p.(A379L)	-	-	Autism and intellectual disability	United Kingdom	McNeill et al. (2020)
*SLC12A2*	c.1229G>A	p.(R410Q)	-	-	Autism and intellectual disability	United Kingdom	McNeill et al. (2020)
*SLC12A2*	c.2675G>A	p.(W892^∗^)	AD	Het	Minor developmental delay. First walked age 3 years	-	McNeill et al. (2020)

Inh, Mode of inheritance, AR, autosomal recessive; AD, autosomal dominant; GT, patient genotype, hom, homozygote, het, heterozygote.

As similarly observed for CLIC5A, the SCL12A2: p.(E979K) mutant protein was found to be relatively expressed in higher proportion when compared to the wild-type (Figure 1C). The p.(E979K) variant is located on exon 21 of *SLC12A2* gene, a region which encodes 17 amino acids critical for the expression and transporter activity in the inner ear (Mutai et al., 2020; Adadey et al., 2021). About 80% of all *SLC12A2* variant associated with hearing impairment are found in the exon 21 of the gene (Adadey et al., 2021). Functional analysis of a splice site variant that results in the skipping of the exon 21 showed a significant reduction of the exon21-skipped variants in the cochlear compared to the wild-type. In fact, the exon21-skipped variant and other variants in exon 21 significantly decreased the chloride transport activity of SLC12A2 protein (Mutai et al., 2020). On the contrary, the SCL12A2: p.(E979K) mutant was proportionally more abundant compared to the wild-type, and this may be due to the variant reducing the protein turnover within the cell.

SLC proteins are predominantly known for their ion transport activities across the cell membrane. This family of proteins are also found throughout cellular organelles which suggests that they perform other intracellular functions aside their predominant cell membrane activity (Pizzagalli et al., 2021). Both SLC12A2: p.(E979K) mutant and wild-type proteins were expressed predominantly in the cytoplasm of the HEK-293 cells. The wild-type SLC12A2 expressing cells were found to have an axon-like cell morphology, with the cell membrane thrown into extended dendrites, a phenotype which was distinctly absent in the mutant p.(E979K) expressing cells. The SLC12 are a family of SLC proteins that are involved in cell volume regulatory activities. These proteins conduct an electroneutral transport of chloride ions with either sodium or potassium ions which results in the movement of water across the cell membrane. The controlled movement of water across the cell membrane regulates cell volume and movement (Turner and Sontheimer, 2014). Inferring from the cell volume and movement activities of the SLC12 family of proteins, it is possible that the mutant SLC12A2: p.(E979K) protein has lost its function and was not able to influence the change in morphology of the HEK-293 cells.

The current study was limited in the fact that it was unable to examine the membrane transport activity of the CLIC5A and SLC12A2 mutant proteins. However, major differences were observed in the localization and morphology of the mutant and wild-type cells from which the biological effect of the variants could be inferred. We recommend that the interaction of all the eight proteins identified to associate with CLIC5A at the base of the stereocilia should be investigated in mice models to elucidate the role of each protein in the formation of the protein-complex and their importance to the structure and function of the stereocilia.

## Conclusion

In this study, we used HEK-293 cell-based experiments to evaluate the pathogenicity of *CLIC5A* [c.224T>C; p.(L75P)] and *SCL12A2* [c.2935G>A: p.(E979K)] variants that were previously reported in the Ghanaian and Cameroonian populations respectively. The protein expression, localization, and cell morphology of the mutant cells were different from their respective wild-type cells showing that the variants likely altered the properties and function of the proteins and thus are pathogenically relevant. Therefore, the study has generated novel biological data associated with the pathogenicity of targeted *CLIC5A* and *SLC12A2* variants, found in two African families, and expands our understanding of their pathobiology in HI.

## Data Availability

The original contributions presented in the study are included in the article/Supplementary Material, further inquiries can be directed to the corresponding author.
