# Supplementary material for: Cell-based analysis of CLIC5A and SLC12A2 variants associated with hearing impairment in two African families
Source: Front Genet. 2022 Aug 11;13:924904. doi: 10.3389/fgene.2022.924904 (PMC9403182; doi:10.3389/fgene.2022.924904)
Supplement: Supplementary file 1 [file DataSheet1.pdf]

## Supplementary Information

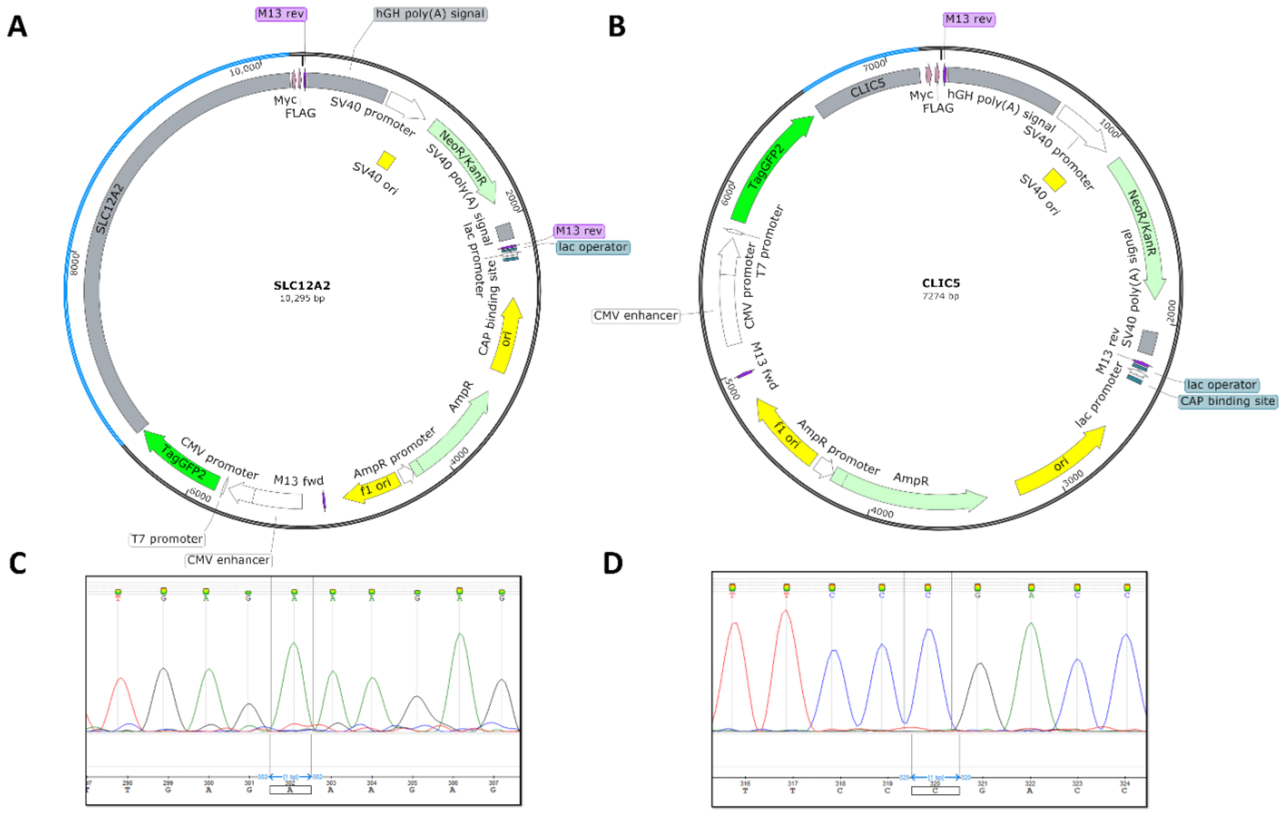


*SLC12A2* SDM primers

| | |
|---|---|
| SLC12A2-SDM-1 | 5`CAATTACACACAAAGTTGAGAAAGAGGATGGCAAGACTGC 3` |
| SLC12A2-SDM-2 | 5`GCAGTCTTGCCATCCTCTTTCTCAACTTTGTGTGTAATTG 3` |

*CLIC5* SDM primers

| | |
|---|---|
| CLIC5-SDM-1 | 5`CACCCGCCCTTCCCGACCTTCAACGGG 3` |
| CLIC5-SDM-2 | 5`CCCGTTGAAGGTCGGGAAGGGCGGGTG 3` |

**Figure S1: Plasmid constructs and chromatogram of *SLC12A2* and *CLIC5* mutants**. Plasmid construct of (A) *SLC12A2* (B) *CLIC5* purchased from ORIGENE (https://www.origene.com/). Using specific primers, site directed mutagenesis (SDM) was used to create (C) c.2935G>A:p.(E979K) mutant *SLC12A2* and (D) c.224T>C; p.(L75P) *CLIC5* plasmids. Both constructs have FLAG and Myc tags at the N-terminus.

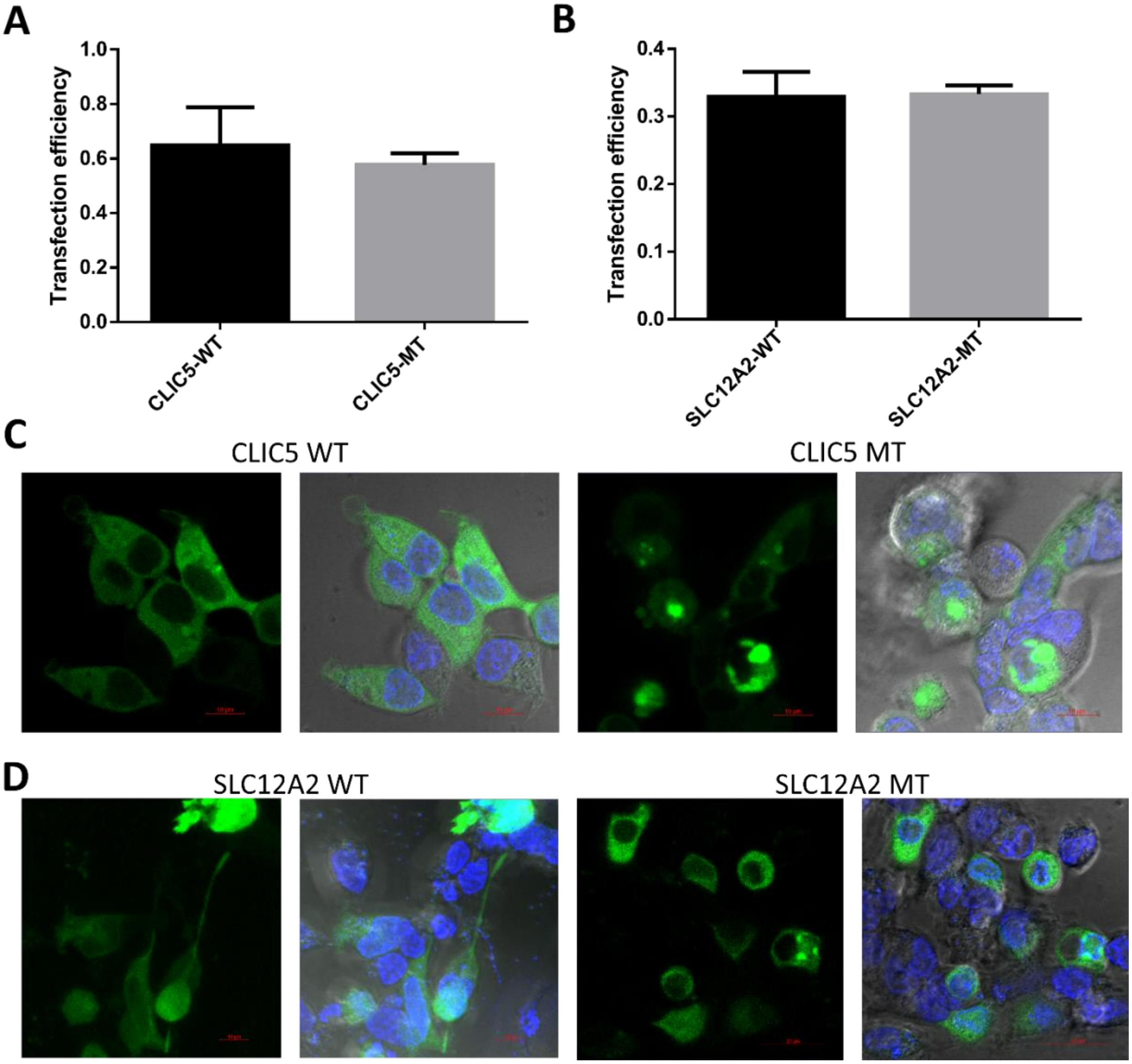


**Figure S2: No statistical difference was observed in the transfection efficiency of the wildtype and mutant cells.** Transfection efficiency of **(A)** CLIC5 and **(B)** SLC12A2 transfected cells. Errors bars represent standard deviation. To calculate the transfection efficiency, the number of cells with the GPF signal was divided by the total number of cells in the selected field. Images showing similar transfection efficiency between the wildtype and mutant **(C)** CLIC5 and **(D)** SLC12A2 transfected cells.

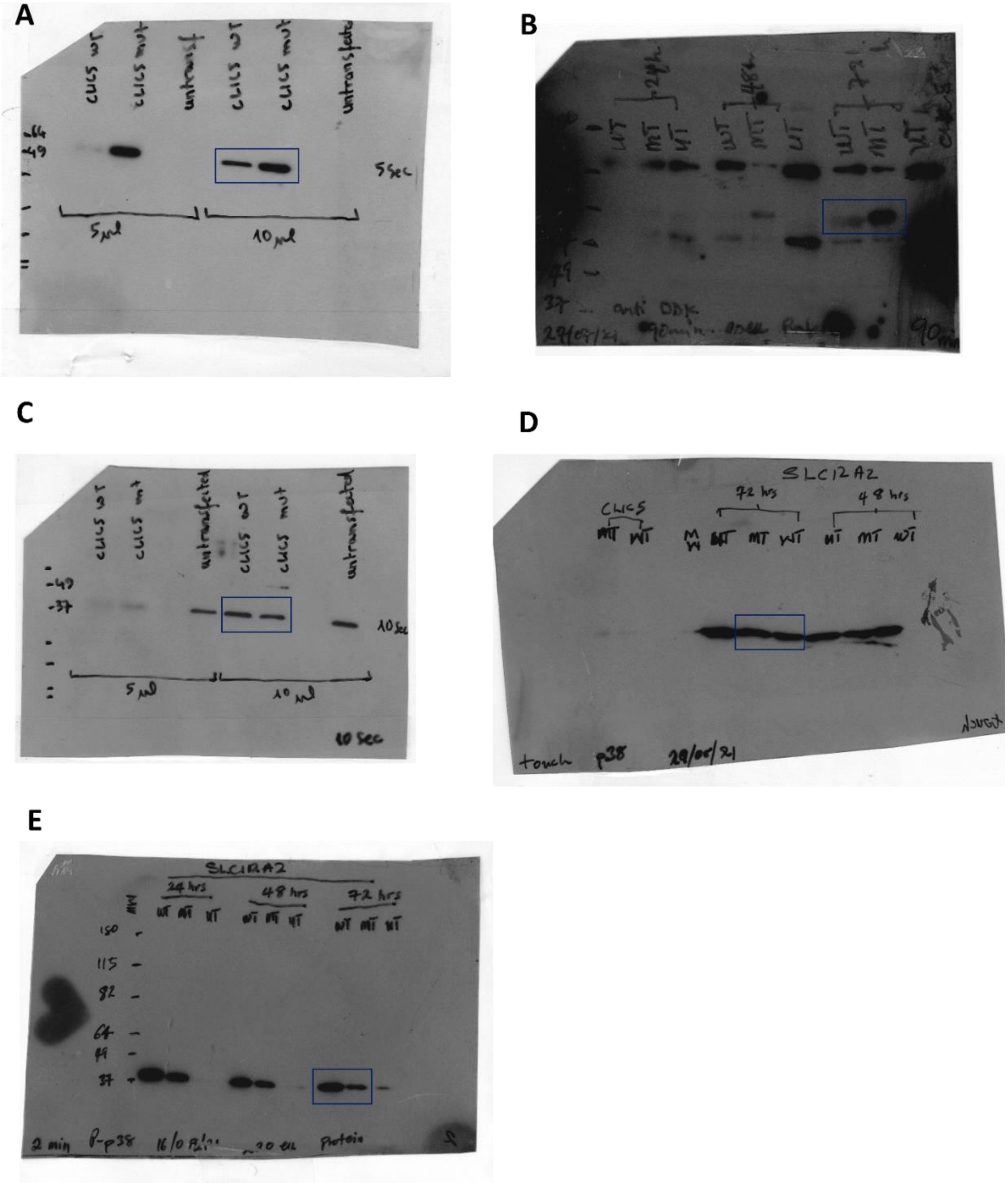


**Figure S3: Images of the uncropped western blots. (A)** CLIC5, **(B)** SLC12A2 **(C)** p38 for CLIC5, **(D)** p38 for SLC12A2 and **(E)** SLC12A2 phosphorylated p38 western blot images. The bands of interest are highlighted with blue rectangles.

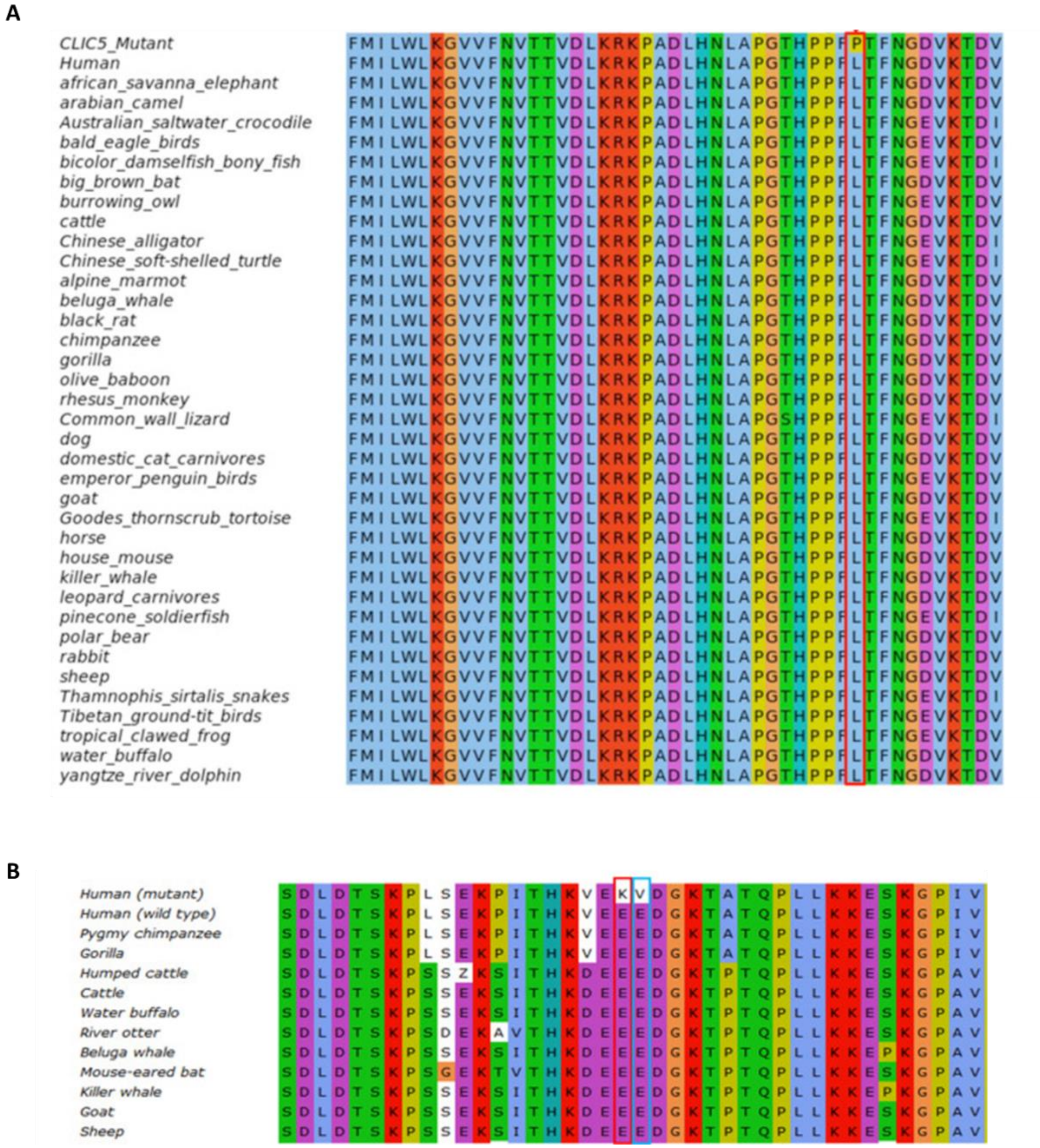


**Figure S4: CLIC5 and SLC12A2 protein sequence alignment**. **(A)** Evolutionary conservation of the CLIC5:p.(L75P) variant position (indicated by the red arrow). **(B)** Protein sequence alignment of SLC12A2. The amino acid position of SLC12A2: p.(E979K) was highlighted with a red rectangle. Please find the details of the CLIC5 [1] and SLC12A2 [2] protein alignments in our previous publications.

## Literature review

To identify peer-reviewed reports that associated *CLIC5* variants with HI and determine the experimental evidence of CLIC5 protein-proteins interactions in the inner ear, a literature search was conducted with the search terms (“CLIC5” AND (“Hearing loss” OR “Hearing impairment” OR “Deafness”)) on PubMed, Africa-Wide Information, and Web of Science databases. Eight publications were retained after screening with their titles and abstracts. In a similar way, the search term (“SLC12A2” AND (“Hearing loss” OR “Hearing impairment” OR “Deafness”)) was used to conduct a literature search on the databases mentioned above for literature on *SLC12A2* association with HI. The search was conducted independently by two reviewers from 1st December 2021 to 6th January 2022. The retrieved publications were independently screened by the two reviewers using the title followed by the abstract. Relevant data were extracted from the retrieved records and a third reviewer, an expert in the field was consulted whenever the two reviewers had a disagreement.

**Table S1: Proteins with experimental evidence of interaction with CLIC5 at the base of stereocilia**

| Protein | Description | Method used to investigate association with CLIC5 | Expressed in the mouse inner ear | Reference |
|---|---|---|---|---|
| Radixin (RDX) | Radixin a deafness associated gene that encodes a protein crucial for binding the barbed end of actin filaments to the plasma membrane. | 1. Immunolocalizations using scanning electron microscopy (SEM)<br>2. Immunoprecipitation/pull-down assay<br>3. Immunohistochemistry | Yes | [3, 4] |
| Ezrin (EZR) | Villin 2 (ezrin) is required for the formation of microvilli and membrane ruffles on the apical pole. Ezr is expressed in the mouse inner ear. | | Yes | [5] |
| GRXCR2 | Glutaredoxin domain-containing cysteine-rich protein 2 (GRXCR2) is expressed in the inner ear and involved in maintaining cochlear stereocilia bundles (important structure for sound detection). | 1. Immunolocalization analyses | Yes | [6] |
| Teperin (TPRN) | Teperin is an autosomal recessive deafness associated gene. | 1. Immunolocalizations using scanning electron microscopy (SEM)<br>2. Immunoprecipitation/pull-down assay | Yes | [3, 6] |
| ACTG1 | Actin, cytoplasmic 2 (ACTG1) is a highly conserved and ubiquitous protein expressed in all eukaryotic cells. Actin is involved in cell motility. | 1. Immunolocalization analyses | Yes | [6] |
| RIPOR2/ FAM65B | Rho family-interacting cell polarization regulator 2 (RIPOR2) is an inhibitor of the small GTPase RHOA and is involved in the regulation of myoblast and hair cell differentiation. RIPOR2 is required for normal inner and outer hair cell stereocilia development. | 1. Immunolocalization analyses | Yes | [6] |
| Myosin VI (MYO6) | Myosin 6 is an actin-based motor molecule that is involved in intracellular movements. | 1. Immunolocalizations using scanning electron microscopy (SEM)<br>2. Immunoprecipitation/pull-down assay | Yes | [3] |
| PTPRQ | Protein tyrosine phosphatase receptor Q (PTPRQ) is | 1. Immunolocalizations using scanning electron microscopy (SEM)<br>2. Immunoprecipitation/pull-down assay | Yes | [3] |

**Table S2: STRING predicted functional partners to CLIC5 and proteins that interact with CLIC5**

| Protein | Description | Expression in mouse inner ear (data from gEAR) | Evidence of association | | | | | |
|---|---|---|---|---|---|---|---|---|
| | | | Co-expression | Experiments | Databases | Text-mining | Homology | Score |
| SLC9A3R1 | Na(+)/H(+) exchange regulatory cofactor NHE-RF1; Scaffold protein that connects plasma membrane proteins with members of the ezrin/moesin/radixin family and thereby helps to link them to the actin cytoskeleton and to regulate their surface expression. | Yes | Yes | Yes | Yes | Yes | - | 0.999 |
| SLC9A1 | Solute carrier family 9 (sodium/hydrogen exchanger), member 1; Sodium/hydrogen exchanger 1; Involved in pH regulation to eliminate acids generated by active metabolism or to counter adverse environmental conditions. Major proton extruding system driven by the inward sodium ion chemical gradient. | Yes | - | Yes | Yes | Yes | - | 0.998 |
| ICAM1 | Intercellular adhesion molecule 1; ICAM proteins are ligands for the leukocyte adhesion protein LFA-1 (integrin alpha-L/beta-2). During leukocyte trans- endothelial migration, ICAM1 engagement promotes the assembly of endothelial apical cups through ARHGEF26/SGEF and RHOG activation; CD molecules | No | Yes | Yes | Yes | Yes | - | 0.998 |
| ACTB | Actin, cytoplasmic 1; Actins are highly conserved proteins that are involved in various types of cell motility and are ubiquitously expressed in all eukaryotic cells | Yes | Yes | Yes | Yes | Yes | Yes | 0.998 |
| CFTR | Cystic fibrosis transmembrane conductance regulator: Epithelial ion channel that plays an important role in the regulation of epithelial ion and water transport and fluid homeostasis. Mediates the transport of chloride ions across the cell membrane. Channel activity is coupled to ATP hydrolysis. The ion channel is also permeable to HCO(3-); selectivity depends on the extracellular chloride concentration. Exerts its function also by modulating the activity of other ion channels and transporters. | No | - | Yes | Yes | Yes | - | 0.997 |
| SLC9A3R2 | Na(+)/H(+) exchange regulatory cofactor NHE-RF2; Scaffold protein that connects plasma membrane proteins with members of the ezrin/moesin/radixin family and thereby helps to link them to the actin cytoskeleton and to regulate their surface expression. Necessary for cAMP-mediated phosphorylation and inhibition of SLC9A3. May also act as scaffold protein in the nucleus; PDZ domain containing | Yes | Yes | Yes | - | Yes | - | 0.997 |

| | | | | | | | | |
|---|---|---|---|---|---|---|---|---|
| ICAM2 | Intercellular adhesion molecule 2; ICAM proteins are ligands for the leukocyte adhesion protein LFA-1 (integrin alpha-L/beta-2). ICAM2 may play a role in lymphocyte recirculation by blocking LFA-1-dependent cell adhesion. It mediates adhesive interactions important for antigen- specific immune response, NK-cell mediated clearance, lymphocyte recirculation, and other cellular interactions important for immune response and surveillance; CD molecules | No | - | Yes | - | Yes | - | 0.997 |
| GIPC1 | PDZ domain-containing protein GIPC1; May be involved in G protein-linked signaling; Belongs to the GIPC family | Yes | Yes | Yes | - | Yes | - | 0.996 |
| CD44 | CD44 antigen; Receptor for hyaluronic acid (HA). Mediates cell-cell and cell-matrix interactions through its affinity for HA, and possibly also through its affinity for other ligands such as osteopontin, collagens, and matrix metalloproteinases (MMPs). Adhesion with HA plays an important role in cell migration, tumor growth and progression. | Yes | Yes | Yes | - | Yes | - | 0.996 |
| VCAM1 | Vascular cell adhesion protein 1; Important in cell-cell recognition. Appears to function in leukocyte-endothelial cell adhesion. Interacts with integrin alpha-4/beta-1 (ITGA4/ITGB1) on leukocytes and mediates both adhesion and signal transduction. | Yes | - | Yes | Yes | Yes | - | 0.992 |

**References**


1. Wonkam-Tingang E, Schrauwen I, Esoh KK, Bharadwaj T, Nouel-Saied LM, Acharya A, et al. Bi-Allelic Novel Variants in CLIC5 Identified in a Cameroonian Multiplex Family with Non-Syndromic Hearing Impairment. Genes (Basel). 2020;11:1-12.
2. Adadey SM, Schrauwen I, Aboagye ET, Bharadwaj T, Esoh KK, Basit S, et al. Further confirmation of the association of SLC12A2 with non-syndromic autosomal-dominant hearing impairment. J Hum Genet. 2021:1-7.
3. Salles FT, Andrade LR, Tanda S, Grati M, Plona KL, Gagnon LH, et al. CLIC5 stabilizes membrane-actin filament linkages at the base of hair cell stereocilia in a molecular complex with radixin, taperin, and myosin VI. Cytoskeleton (Hoboken). 2014;71:61-78.
4. Gagnon LH, Longo-Guess CM, Berryman M, Shin J-B, Saylor KW, Yu H, et al. The chloride intracellular channel protein CLIC5 is expressed at high levels in hair cell stereocilia and is essential for normal inner ear function. Journal of Neuroscience. 2006;26:10188-98.
5. Bretscher A, Reczek D, Berryman M. Ezrin: a protein requiring conformational activation to link microfilaments to the plasma membrane in the assembly of cell surface structures. Journal of cell science. 1997;110:3011-18.
6. Li J, Liu C, Zhao B. N-Terminus of GRXCR2 Interacts With CLIC5 and Is Essential for Auditory Perception. Front Cell Dev Biol. 2021;9:1-9.
